# Efficacy and safety of Shenling Atractylodes Powder in the treatment of ulcerative colitis: A protocol for systematic review and meta-analysis

**DOI:** 10.1097/MD.0000000000025355

**Published:** 2021-04-09

**Authors:** Quanhui Zhang, Yongwen Deng, Jinlong Wang, Feihong Haung, Yiduo Zhou, Mingyan Jia, Haoran Yi

**Affiliations:** a Affiliated Hospital of Jiangxi University of Traditional Chinese Medicine; b School of Clinical Medicine, Jiangxi University of Traditional Chinese Medicine, Nanchang 330006, China.

**Keywords:** Shenling Atractylodes Powder plus or minus, systematic review and meta-analysis, ulcerative colitis

## Abstract

**Background:**

Ulcerative colitis (UC) is a kind of chronic non-specific ulcerative colitis, which is characterized by repeated abdominal pain, diarrhea, and mucus purulent stool. The disease is more recurrent, easy to delay, and canceration, seriously affect the quality of life, increase the economic burden of patients and society, treatment is more difficult, the World Health Organization as one of the modern refractory diseases. Shenling Atractylodes Powder in the treatment of ulcerative colitis showed a strong advantage, the effect is accurate. Therefore, this paper will systematically evaluate and meta-analyze the efficacy and safety of heat-sensitive moxibustion in the treatment of ulcerative colitis.

**Methods:**

Eight electronic databases were searched, including the PubMed, Embase, Web of Science, Cochrane Library, China National knowledge Infrastructure, China Science and Technology Journal Database, Wanfang Database, and China Biomedical Literature Database. We will search in the above electronic database from early 2021 to December without any language restrictions. Outcome indicators, including colonic mucosal symptom score Mayo colonoscopy grading, total effective rate, total incidence of adverse reactions, clinical symptom score, recurrence rate, laboratory indicators: IL-6, IL-9, TNF-α, IL-4, IL-10 inflammation-related factor levels. Rev Man5.3 software will be used for statistical analysis. The efficacy and safety results of Shenling Atractylodes Powder in the treatment of ulcerative colitis will be used as the average difference between the risk ratio of dichotomy data and the 95% co-card interval of continuous data.

**Results:**

When this research program is completed, the relevant results can be obtained.

**Ethics and dissemination:**

This article does not need to pass the ethics committee review, because this article does not involve the ethics question, only collates the related literature research. The results of this study will be disseminated in the form of a paper to help better guide the clinical practice of heat-sensitive moxibustion in the treatment of ulcerative colitis.

**Registration Number:**

INPLASY202120018.

## 1 Introduction

Ulcerative colitis is a chronic non-specific inflammatory bowel disease characterized by long-term recurrent abdominal pain, diarrhea, and mucus purulent stool. The site of the disease is limited to the colorectal mucosa and submucosa, the colon mucosa is diffuse, continuous chronic inflammatory, ulcerative changes, is the cause of the disease, the mechanism of the disease is not clear, it is considered to be related to immunity, heredity, environment, intestinal microenvironment, mental factors, and so on.^[1]^ The disease is more recurrent and cannot be cured, and may be cancerous, treatment is more difficult, the World Health Organization as one of the modern refractory diseases.^[2]^ The prevalence of ulcerative colitis in China is about 11.6/105.^[3]^ However, with the change of living habits, diet structure, living standard, environment, and diagnostic techniques, the incidence of ulcerative colitis in China is increasing year by year.^[4,5]^ It causes great pain and physical and mental exhaustion to the patients, seriously reduce the standard of living, and increase the financial burden of the family.^[6]^ According to the Consensus on the Diagnosis and Treatment of Inflammatory Bowel Disease, which was formulated in Beijing, China in 2018, the individual treatment plan with 5-ASA (5-aminosalicylic acid), hormones, and immunosuppressants was formulated after comprehensive evaluation of the patient's condition, and its clinical efficacy was still good.^[6]^ However, 5-ASA may also induce colitis, complications such as pericarditis and hepatitis.^[7]^ Glucocorticoids are effective in many patients with moderate and severe ulcerative colitis, but many patients still have glucocorticoid resistance or glucocorticoid dependence. In recent years, biological agents such as Infliximab, Vidoxizumab, and Vexizumab have become popular drugs in the treatment of ulcerative colitis. In recent years, the clinical effect of Shenling Atractylodes Powder in the treatment of ulcerative colitis has been reported one after another.^[8–13]^ But evidence-based medicine is lacking. Therefore, the purpose of this study was to explore the clinical efficacy and safety of Shenling Atractylodes Powder in the treatment of ulcerative colitis through systematic evaluation and meta-analysis.

## 2 Method

### 2.1 Study registration

The Protocol was registered on 5 February 2021 at the International Registration System Review and Meta-Analysis Protocol Platform (INPLASY) and last updated on 5 February 2021 (registration number INPLASY202120018).

### 2.2 Inclusion criteria for research selection

#### 2.2.1 Type of study

The clinical randomized controlled trial (RCTs) included Shenling Atractylodes Powder and ulcerative colitis (UC), the publication state is unlimited, the language is Chinese or English.

#### 2.2.2 Types of participants

Have clear and recognized diagnostic criteria and efficacy criteria, and all patients were diagnosed UC, there are no restrictions on the sex, age, occupation, course of disease, source of cases.

#### 2.2.3 Types of interventions

##### 2.2.3.1 Experimental intervention

Oral Shenling Atractylodes Powder plus or minus prescription therapy, or based on oral Shenling Atractylodes Powder plus or minus prescription will also be included.

##### 2.2.3.2 Control interventions

The control group will receive one of the following treatments: routine medication, non-treatment, and placebo.

### 2.3 Types of outcome measures

#### 2.3.1 Main results

Colonic mucosal symptom score, total effective rate, clinical symptom score, recurrence rate Mayo enteroscopy grading.

##### 2.3.1.1 Secondary outcomes

IL-6, IL-9, TNF-α, IL-4, IL-10, the level of inflammatory related factors, and the total incidence of adverse reactions.

### 2.4 Exclusion criteria

Repetitive literature (including the same literature published in different journals or conference papers, the same literature published in Chinese and English journals, and the same original data); incomplete literature with incomplete results data; unclear diagnostic criteria; and evaluation criteria for efficacy.

### 2.5 Search methods and strategies for this study

#### 2.5.1 Electronic database retrieval

We will search 8 electronic databases, including PubMed, Embase, science network, Cochrane Library, China National Knowledge Infrastructure, China Science and Technology Journal Database, Wanfang Database, and China Biomedical Literature Database. We will search in the above electronic database from early 2021 to December without any language restrictions and the subject words were combined with free words to search the relevant literature. Search terms included disease (“ulcerative colitis” or “idiopathic rectoconjunctivitis” or “ gradient colitis” or “inflammatory bowel disease”) and intervention (“Shenling Atractylodes Powder” or “Shenling Atractylodes Powder plus or minus”) and study type (“randomized controlled trial” or “ controlled clinical trial” or “randomized trial” or “RCT”). PubMed search strategy proposed is shown in Table 1.

**Table 1:** Search strategy (PubMed).

Number	Search terms
#1	MeSH: “Shenling Atractylodes Powder”
#2	Ti/Ab: “Shenling Atractylodes Powder” OR “Shenling Atractylodes Powder plus or minus”
#3	#1 OR #2
#4	MeSH: “Colitis, ulcerative”
#5	Ti/Ab: “ulcerative colitis” OR “idiopathic rectoconjunctivitis” OR “gradient colitis” OR “inflammatory bowel disease” OR “mammary glands”
#6	#4 OR #5
#7	MeSH: “randomized controlled trial” OR “randomized controlled trial as Topic” OR “controlled clinical trial”
#8	Ti/Ab: “randomized controlled trial” OR “controlled clinical trial” OR “randomized”
#9	#7 OR #8
#10	#3 AND #6 AND #9

Ab = abstract, MeSH = medical subject headings, Ti = title.

#### 2.5.2 Search for other resources

The reference list of this study will be combined with the manual retrieval of literature resources to search for relevant conference papers that meet the inclusion criteria. furthermore, ongoing and recently completed studies, as well as grey literature, will be searched on the Clinicaltrials.gov.

### 2.6 Data extraction and management

#### 2.6.1 Literature inclusion and data extraction

**Figure 1:**
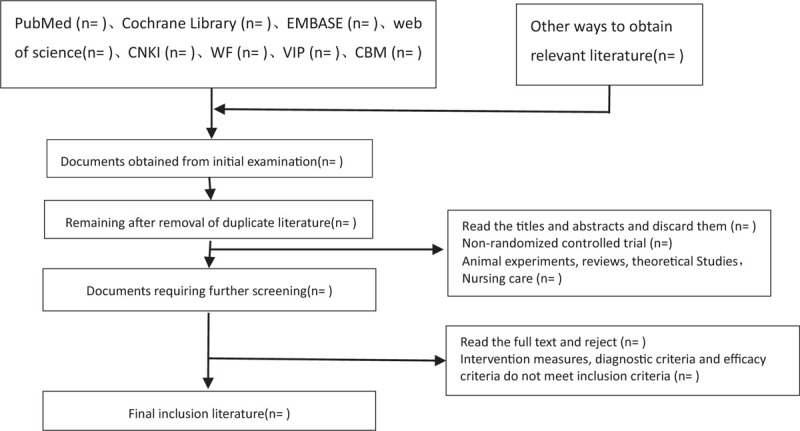
Flow chart of literature incorporation.

The 2 researchers independently read the title and abstract of the literature we obtained, read the full text of the trials that might meet the inclusion criteria to determine whether the inclusion criteria were truly met, and discussed the conflicting literatures or let the third researcher decide whether to include them. Two researchers independently extracted data from the included studies, including study design, intervention measures and methods, measurement indicators, results, methodological contents such as hidden grouping and blind method, etc, and a third evaluator checked the consistency of the data. If the required information is incomplete, we will contact the original author for the required data. The inclusion process of this study will be carried out as shown in Figure 1.

#### 2.6.2 Methodological quality evaluation

Two evaluators independently select the literature according to the inclusion and exclusion criteria and crosscheck. In case of disagreement, a third evaluator will assist in the decision. The extracted data included the first author, year of publication, number of patients, age, gender, intervention measures, outcome indicators, etc. The Jadad scale to evaluate quality into literature, including: random sequence (right 2 points, 1 point not clear, inappropriate 0), distribution, hidden (right 2 points, 1 point not clear, inappropriate 0), blinded (right 2 points, 1 point not clear, inappropriate 0), lost to follow-up and exit (describe 1 point, not describe 0); 0 to 3 is classified as low quality and 4 to 7 as high quality.

### 2.7 Statistical analysis

#### 2.7.1 Quantitative data synthesis

Meta-analysis will be performed using Rev Man5.3.0 software. The odds ratio (OR) and its 95% confidence interval (CI) will be used as the counting data, while the weighted mean difference (WMD) and its 95% CI will be used as the measurement data.

#### 2.7.2 Assessment of heterogeneity

The heterogeneity test will be carried out first among all studies, *I*^2^ test will be used. When *P* > .1 and *I*^2^ < 50%, the fixed effect model will be used; otherwise, the random effect model will be used. When the clinical heterogeneity between the 2 studies is large, only descriptive analysis will be performed.

#### 2.7.3 Publication bias

When the number of qualified RCTs is sufficient, we will use the inverted funnel Egger to test the potential publication bias.

#### 2.7.4 Subgroup analysis

If there are enough data, we plan to conduct a subgroup analysis for different groups, including different comparators, treatment times, sorts of western medicine, quality of evidence, and outcome measures.

#### 2.7.5 Sensitivity analysis

The goal of sensitivity analysis is to identify the sources of heterogeneity and confounding factors.

If the trials’ data is sufficient, a sensitivity analysis will be performed by excluding the low-quality or high-weight studies one by one.

## 3 Discussion

According to TCM, UC belongs to the category of dysentery, diarrhea, red and white dysentery.^[14]^ The main reason for the UC is that the spleen is not healthy and the dampness poison runs through the whole process of the disease.^[15]^ Therefore, invigorating spleen and removing dampness is the basic treatment of this disease. Shenling Atractylodes Powder can strengthen spleen and invigorate Qi and clear heat and dampness. Modern pharmacological studies have found that total glucosides of Radix Paeoniae Alba in Shenling Atractylodes Powder plus and reducing prescription can effectively inhibit inflammatory cell infiltration in UC intestinal mucosa, alleviate mucosal injury, reduce the content of inflammatory factors such as IFN-γ, IL-5, IL-6 in serum of UC rats, increase the content of inflammatory factor IL-4, IL-10, and relieve UC symptoms.^[16,17]^ The main component of Fructus Aurantii is hesperidin, which can effectively reduce colon mucosal injury, significantly reduce the ratio of colon weight to colon length, significantly reduce the DAI, of mice to down-regulate MPO activity and MDA content in colon tissue, and down-regulate the expression of serum IL-6.^[18]^ In recent years, due to its remarkable curative effect, this treatment method has been paid more and more attention in clinic, but there is still a lack of systematic evaluation of the effectiveness and safety of Shenling Atractylodes Powder UC treatment. Therefore, it is necessary to systematically evaluate the UC of Shenling Atractylodes Powder in this study, and provide evidence-based medical evidence for the clinical guidance of Shenling Atractylodes Powder in the treatment of UC. Most trials describe complementary and alternative drugs for acupuncture treatment.

However, this study has several limitations. First, although we have collected a large amount of literature through comprehensive search strategies of different databases without any language restrictions, we cannot be sure that all relevant RCT will be included. Secondly, limited by the retrieval conditions, only the Chinese and English databases will be retrieved, resulting in a certain language deviation. Third, we may have difficulty retrieving raw data from published sources because the corresponding author may not produce raw data.

## Author contributions

**Data curation:** Jinlong Wang.

**Formal analysis:** Yiduo Zhou.

**Investigation:** Feihong Haung, Mingyan Jia.

**Methodology:** Yiduo Zhou, Haoran Yi.

**Project administration:** Quanhui Zhang.

**Software:** Feihong Haung, Mingyan Jia.

**Supervision:** Yiduo Zhou, Haoran Yi.

**Validation:** Yongwen Deng.

**Visualization:** Yongwen Deng, Jinlong Wang.

**Writing – original draft:** Quanhui Zhang, Jinlong Wang.

**Writing – review & editing:** Quanhui Zhang, Jinlong Wang.
